# Analysis of Combined Transcriptomes Identifies Gene Modules that Differentially Respond to Pathogenic Stimulation of Vascular Smooth Muscle and Endothelial Cells

**DOI:** 10.1038/s41598-017-18675-2

**Published:** 2018-01-10

**Authors:** Xiaokang Pan, Bowen Wang, Tiezheng Yuan, Mengxue Zhang, K. Craig Kent, Lian-Wang Guo

**Affiliations:** 1 Department of Surgery, Wisconsin Institute for Medical Research, University of Wisconsin School of Medicine and Public Health, Madison, WI 53705 USA; 2 James Molecular Laboratory, Comprehensive Cancer Center, The Ohio State University, Columbus, OH 43240 USA; 3 Department of Surgery, College of Medicine, The Ohio State University, Columbus, OH 43210 USA; 4 Department of Surgery and Department of Physiology & Cell Biology, Davis Heart and Lung Research Institute, The Ohio State University, Columbus, OH 43210 USA

## Abstract

Smooth muscle cells (SMCs) and endothelial cells (ECs) are vital cell types composing the vascular medial wall and the atheroprotective inner lining, respectively. Current treatments for cardiovascular disease inhibit SMC hyperplasia but compromise EC integrity, predisposing patients to thrombosis. Therapeutics targeting SMCs without collateral damage to ECs are highly desirable. However, differential (SMC versus EC) disease-associated regulations remain poorly defined. We conducted RNA-seq experiments to investigate SMC-versus-EC differential transcriptomic dynamics, following treatment of human primary SMCs and ECs with TNFα or IL-1β, both established inducers of SMC hyperplasia and EC dysfunction. As revealed by combined SMC/EC transcriptomes, after TNFα or IL-1β induction, 174 and 213 genes respectively showed greater up-regulation in SMCs than in ECs (SMC-enriched), while 117 and 138 genes showed greater up-regulation in ECs over SMCs (EC-enriched). Analysis of gene interaction networks identified central genes shared in the two SMC-enriched gene sets, and a distinct group of central genes common in the two EC-enriched gene sets. Significantly, four gene modules (subnetworks) were identified from these central genes, including SMC-enriched JUN and FYN modules and EC-enriched SMAD3 and XPO1 modules. These modules may inform potential intervention targets for selective blockage of SMC hyperplasia without endothelial damage.

## Introduction

Cardiovascular disease has long remained the leading cause of death and morbidity in developed countries^1^. Implantation of a drug-eluting stent is the most commonly performed coronary intervention that ameliorates the symptoms of cardiovascular disease^2^. However, late stent thrombosis and in-stent restenosis remain critical problems that lead to stroke, myocardial infarction, or sudden death^3^.

Smooth muscle cells (SMCs) and endothelial cells (ECs) are major cell types in the vascular wall constituting the media layer and the inner lining of endothelium, respectively. Disturbation of their homeostasis due to surgical interventions often leads to failure of those treatments. After surgical injuries, SMCs undergo a hyperplastic transformation and form neointimal lesions re-narrowing the lumen (restenosis)^4^. In the meanwhile, damaged ECs no longer function as the protective barrier between SMCs and pathogenic stimulants (or cells) in the circulation^5^. Instead, they transform into a pro-inflammatory and pro-coagulant phenotype, promoting thrombosis as well as restenosis^6^.

Because of a close vicinity in the vascular wall, SMCs and ECs are exposed to similar environments of pathophysiological stimuli, yet their responses are drastically different. These differential responses profoundly influence the outcomes of surgical (and pharmacological) interventions using drug-eluting stents. Whereas SMC hyperplasia, i.e., proliferation, migration, and inflammation, produces neointimal lesions, reendothelialization (EC re-growth) is critically important for preventing thrombosis and attenuating restenosis^4,5^. To date, the only therapeutics applied in drug-eluting stents are sirolimus (or analogs) and paclitaxel^6–9^. While these anti-proliferative drugs are effective inhibitors of SMC hyperplasia, they are toxic to ECs and retard reendothelialization, predisposing patients to thrombosis and restenosis^10^. Thus, endothelium-protective agents that selectively inhibit SMC hyperplasia are urgently needed^11^. Unfortunately, thus far reports of such agents have been scarce. In particular, genomewide investigations of transcriptomes or pathways differentially regulated in SMCs versus ECs in response to pathogenic stimuli are extremely rare.

In this study, we performed RNA sequencing (RNA-seq) and global analyses of differential SMC-versus-EC transcriptomic responses, to the same pathogenic cytokine stimulant under stringently controlled conditions. The objective was to identify the gene modules (or subnetworks) that are highly up-regulated in SMCs yet little affected or regulated toward the opposite direction in ECs, so that future interventions targeting these differential regulations would effectively mitigate SMC hyperplasia yet minimally disturb EC homeostasis. We were able to extract four gene modules with distinct pathophysiological roles (evidenced in the literature) in SMCs and ECs, which could inform potential targets for intervention to selectively inhibit neointimal development without adverse effects on reendothelialization.

## Methods

### Cell culture

Human aortic SMCs and ECs were purchased from Lonza, and cultured at 37 °C with 5% CO_2_ in their respective optimal media (SmGM-2 with 5% FBS and EGM-2 with 2% FBS, Lonza). For cell culture expansion, 0.25% Trypsin was used for detachment of SMCs, while Accutase (Lifetechnologies, Carlsbad, CA) was used for ECs. Cells between passage 5 and 7 were used for all experiments. For cytokine stimulation, cells at ~70% confluency were starved overnight in basal medium containing 0.5% fetal bovine serum (FBS) and then treated for 4 h with recombinant TNF-α or IL-1β (R&D Systems, MN) at a final concentration of 20 ng/mL. The same experiments without adding TNFα or IL-1β were conducted as solvent control. Three replicate experiments were conducted for each cytokine treatment or control condition.

### RNA extraction and purification, library preparation, and RNA sequencing

RNA-seq was conducted at the Penn State College of Medicine Genome Sciences and Bioinformatics Core Facility, following recently published methods^12^ with modifications. Briefly, total RNAs were isolated from SMCs or ECs using TRIzol reagents (Invitrogen) followed by confirming high quality of purified mRNAs; the A260/A280 ratio was determined to be >1.9 by NanoDrop (Thermo Scientific), and the RNA integrity number was measured to be >7 using BioAnalyzer RNA 6000 Nano Kit (Agilent). The cDNA libraries were prepared using SureSelect Strand Specific RNA Library Preparation Kit (Agilent) and unique barcode sequences were incorporated in the adaptors for multiplexed high-throughput sequencing. The size distribution and concentration of the final product were assessed using BioAnalyzer High Sensitivity DNA Kit (Agilent) and Kapa Library Quantification Kit (Kapa Biosystems). The libraries were then pooled and diluted to 2 nM in EB buffer (Qiagen). Before loading to an Illumina HiSeq. 2500 sequencing analyzer (with TruSeq SR v3 flow cells), the libraries were denatured and diluted to 10 pM in pre-chilled hybridization buffer. After 50 cycles using a single-read recipe (TruSeq SBS Kit v3, Illumina), de-multiplexed sequencing reads (fastq files) that passed the default purify filter were obtained using the Illumina CASAVA pipeline (version 1.8).

### Human genome mapping and analysis of differential gene expression

To analyze the RNA-seq data from human SMCs and ECs, we used HG19 (human genome version 19, UCSC) as the human reference genome. A Bowtie 2^13^ index was built, and TopHat2^14^ was used to align transcripts to the annotated human genome, and transcriptomes were then assembled using Cufflinks 2.0^15^.

The Cuffdiff program in the Cufflinks2.0 package was applied to calculate gene expression values (FPKM) in each sample. For each gene, the FPKM values of three replicate samples were averaged. Then, the fold changes (FC), log2 (FC), p-values and q-values (adjusted p-values) were also computed. Paired t-test of averaged FPKM values showed a significant difference of global gene expression between SMCs and ECs (p-value = 0.01739) under the basal condition without cytokine stimulation.

**Figure 1:**
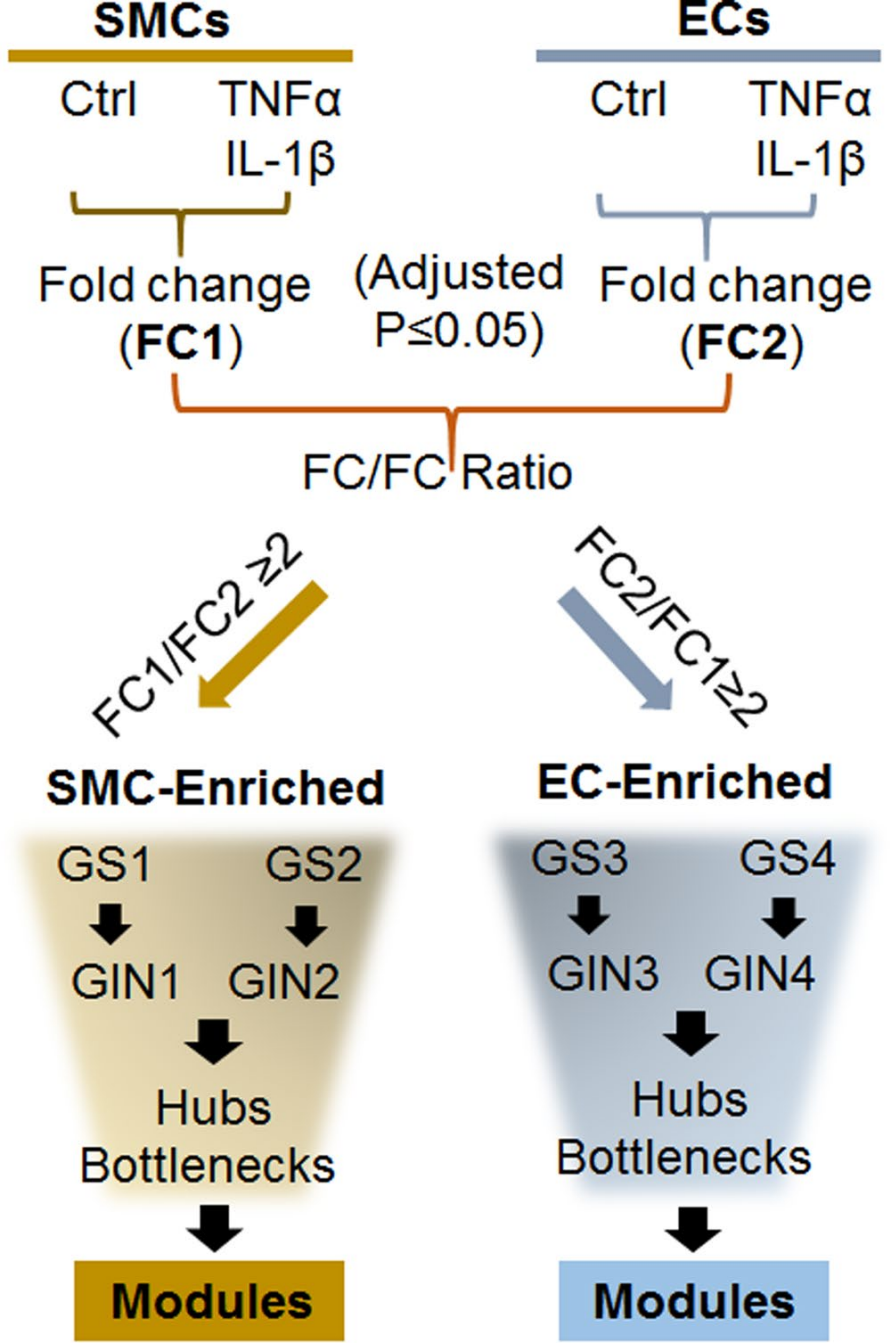
Schematic work flow for identification of gene modules differentially regulated in SMCs and ECs via analysis of combined transriptomes. Gene expression fold change (FC, stimulation versus non-stimulation) was calculated as described in Methods. Gene sets (GS1-GS4) were selected based on the SMC/EC (or reversed) ratios of cytokine-stimulated fold changes (FC1/FC2 or FC2/FC1). The four GSs were used to construct respective GINs, wherein common (in two GSs corresponding to two cytokines) hubs and bottlenecks were identified, from which common gene modules were extracted.

For each gene, the cytokine-stimulated fold change of gene expression in SMCs (FC1) and that in ECs (FC2) were calculated using the FPKM value from cytokine-treated cells versus that from untreated control cells (see Fig. 1). The genes with adjusted p-value ≤ 0.05 in both SMCs and ECs were selected for further analysis. To identify the genes that underwent greater cytokine-stimulated expression fold changes in SMCs versus ECs (or ECs versus SMCs), we analyzed SMC and EC combined transcriptomes. We calculated the FC1/FC2 (and FC2/FC1) ratio for each gene, and selected genes with a ratio ≥2 (see Fig. 1). Thus, these selected genes were considered SMC-versus-EC (or EC-versus-SMC) differentially regulated. They were classified into four gene sets, GS1, GS2, GS3, and GS4 (listed in Tables S2 and S3). Gene clustering was performed using Gene Cluster 3.0^16^ and a heatmap was drawn by Java TreeView^17^ using Log2 (FPKM) values of these genes.

### Pathway and gene ontology (GO) term enrichment data fetch

Pathway and gene ontology enrichment were analyzed *via* DAVID^18^. The gene lists from GS1, GS2, GS3 and GS4 were uploaded with OFFICIAL_GENE_SYMBOL and species Homo sapiens being selected at DAVID website https://david.ncifcrf.gov/summary.jsp, respectively. Pathway and gene ontology enrichment data were downloaded from KEGG_Pathway and GOTERM_BP_FAT categories.

### Gene interaction network (GIN) construction and associated parameter calculation

Web-based program esyN was used to construct GINs. Gene lists from GS1, GS2, GS3 and GS4 were uploaded onto the web server at http://www.esyn.org/. Then, organism H. sapiens (HumanMine) was selected to retrieve gene interaction information from HumanMine database and build gene interaction networks (GINs). When there were small networks appearing along with a large one at the same page, we deleted the small ones using the edit tools built-in with esyN program. After adjusting the layout of the remaining network to the best viewing status, we exported the graphical network as a PNG file.

In a GIN, a hub was defined as a gene (refer to a node in the GIN) has three or more interactions (edges in the GIN). Bottlenecks were defined as the top 10% of the nodes in the high-confidence protein interactome ranked by betweenness centrality (the fraction of all the shortest paths in an interactome that pass through a given node)^19^.

## Results

To investigate differential transcriptomic changes in SMCs and ECs after pathogenic stimulation, we used a well-established cellular model of cytokine induction. TNFα and IL-1β are well-documented potent pathogenic cytokines that stimulate SMC hyperplasia and restenosis^20^ and induce endothelial dysfunction and thrombosis^21,22^. We treated primary human aortic SMCs and ECs with TNFα and then used these cells for RNA-seq. In parallel, we used IL-1β as another pathogenic stimulant to confirm the results from the TNFα experiments. Expeiments were performed as triplicate for each cell type treated with a cytokine.

### A strategy for analysis of SMC/EC combined transcriptomes

The overall strategy for RNA-seq data analysis is diagrammed in Fig. 1. For each cell type (SMC or EC), we calculated gene expression fold change (FC)(cytokine-stimulated versus non-stimulated). We then calculated the ratio of FCs between the two cell types, i.e., SMC-FC versus EC-FC (or reversed) of the same gene. The selection of genes with a FC/FC ratio ≥ 2 resulted in four gene sets (GS1-GS4). The four GSs were used to construct their respective gene interaction networks (GINs), wherein common hub genes and bottleneck genes were identified. We then further extracted gene modules (subnetworks with more centrality) from the common hubs and bottlenecks in each cell type. In this study, a hub is defined as a node (or gene) that has three or more interactions in a GIN. Bottlenecks are the top 10% of the nodes ranked by betweenness centrality in the high-confidence protein interactome^19,23^. Thus, hubs and bottlenecks are independently defined and identified. They may overlap as both are central genes, but are not necessarily related. Detailed data analyses are presented below.

### Analysis of gene expression fold changes following pathogenic stimulation of each cell type (SMC or EC)

As described in the Method section, we calculated gene expression values under each experimental condition, and used these values to calculate cytokine-stimulated (versus non-stimulated) expression fold changes (FCs) in each cell type (SMC or EC), and associated p-values and adjusted p-values. By calculating Spearman correlation on log2^(FC)^, we found that the transcriptomes in the two cell types (without cytokine stimulation) are globally correlated (r = 0.3279, p-value < 2^2e−16^).

To identify genes oppositely regulated in SMCs and ECs following pathogenic cytokine stimulation, we selected up- or down-regulated genes using a conventional cutoff of FC ≥ 2 (up) or FC≤ 0.5 (down) and an adjusted p-value ≤ 0.05. Venn diagrams show that after treatment with TNFα (Figure S1A) or IL-1β (Figure S1B), 5 and 13 genes, respectively, were up-regulated in SMCs but down-regulated in ECs. Four of the 5 genes in the TNFα group were found among the 13 genes in the IL-1β group. In addition, in response to TNFα and IL-1β, respectively, 5 and 6 genes were up-regulated in ECs but down-regulated in SMCs (Table S1).

### Analysis of combined transcriptomes identifies gene sets (GSs) differentially regulated in SMCs versus ECs following pathogenic stimulation

**Figure 2:**
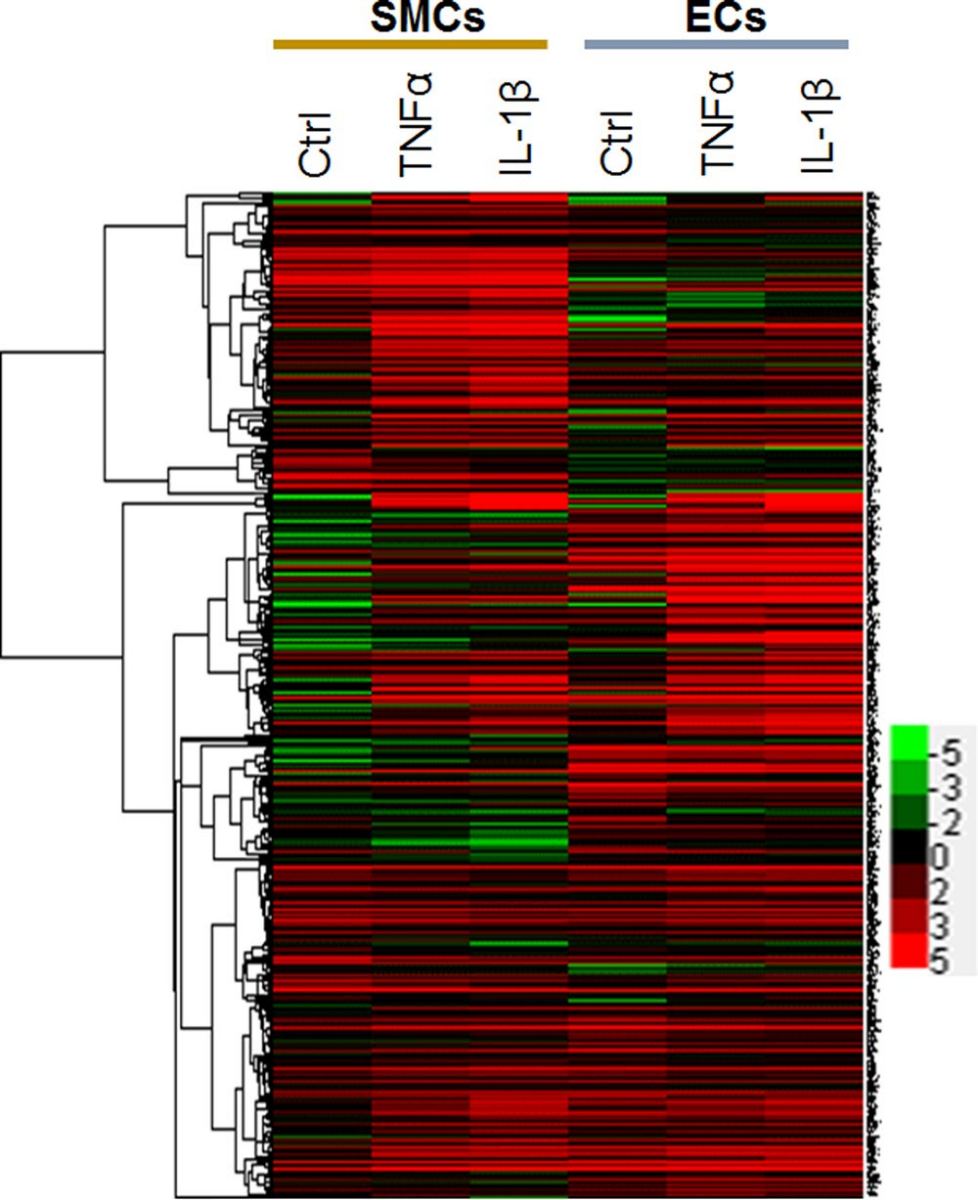
Heatmap of expression values of all genes in the selected four gene sets (GSs). Human SMCs and ECs were cultured, and treated without or with TNFα (or IL-1β), prior to sample preparation for RNA-seq, as described in detail in Methods. Total six conditions (triplicate for each condition): SMCs without cytokine treatment (Ctrl); SMCs treated with TNFα; SMCs treated with IL-1β; ECs without cytokine treatment (Ctrl); ECs treated with TNFα; ECs treated with IL-1β. Gene expression fold changes (stimulation versus non-stimulation) in SMCs (FC1) and in ECs (FC2) were analyzed, and the genes that did not meet the standard of P ≤ 0.05 were excluded. The ratio of fold changes between SMCs and ECs (FC1/FC2 or FC2/FC1) was then calculated. Genes with an FC/FC ratio ≥2 were classified into 4 gene sets (listed in Tables S2 and S3). Their expression values of Log2(FPKM) under the six experimental conditions were used for generating a heatmap with Java TreeView^53^ (value range indicated by the color bar).

To further investigate the functional relationships of differentially regulated genes, we analyzed SMC/EC combined transcriptomes. Unlike the conventional method of analyzing gene expression in a single cell type, this approach allowed us to compare the expression changes of the same gene in two different cell types (SMCs and ECs) responding to the same cytokine stimulation. Only the genes with an adjusted p-value ≤ 0.05 in both SMCs and ECs were included in this analysis. For each gene, we used the cytokine-stimulated fold change in SMCs (FC1) and the fold change in ECs (FC2) to calculate the ratio of FCs between two cell types, in other words, “fold change of fold changes” (i.e., FC1/FC2 or FC2/FC1, see Fig. 1). The genes with an FC/FC ratio ≥ 2 were selected, and classified into four gene sets (GSs) as listed in Tables S2 and S3. GS1 (174 genes, TNFα stimulation) and GS2 (213, IL-1β) were up-regulated in SMCs but down-regulated or slightly up-regulated in ECs (hereafter denoted as SMC-enriched); GS3 (117, TNFα) and GS4 (138, IL-1β) were up-regulated in ECs but down-regulated or slightly up-regulated in SMCs (EC-enriched). Consistent with previously reported similar stimulation effects of TNFα and IL-1β on SMC (or EC) cellular physiology^24–27^, GS1 overlaps GS2 with 105 genes, and GS3 overlaps GS4 with 66 genes. As further shown on the heatmap (Fig. 2) of expression values of all genes in the four gene sets, there were similar patterns of TNFα and IL-1β induced gene expression changes in the same SMC or EC cell type. However, a difference between the two cell types was obvious.

For the selection of genes that showed a high SMC-FC/EC-FC ratio of cytokine-stimulated fold changes yet with a low p-value, we plotted the ratios against log10 p-values (volcano plots, Figure S2). The top 20 genes selected from the upper right corner of the plot were all found in the afore-identified corresponding gene set. This suggests that the volcano plots could be used to visualize the genes that respond to TNFα or IL-1β stimulation with prominently differential (SMC versus EC) changes in expression levels.

### Analyses of pathway and gene ontology (GO) enrichment implicate functional connections of the genes in each gene set

Analysis using the four gene sets (GS1, GS2, GS3, GS4) produced four respective lists of pathways scored by Fisher exact p-values. Approximately 40% of the genes from each GS were included in the enriched pathways listed in Table S4. Validating our sequencing experiments and algorithms, the cytokine signaling pathway scored high in all 4 GSs. Moreover, pathways related to SMC proliferation, migration and inflammation (e.g., MAPK, Toll like and NOD-like receptors) were ranked on the top in GS1 and GS2. Of note, the TGFβ signaling pathway, a known critical regulator in ECs^28^, was highly scored for GS4, an EC-enriched gene set.

We also generated four lists of gene ontology (GO) terms enriched with the genes in GS1, GS2, GS3, and GS4 respectively, and ranked them based on Fisher exact p-values (Figure S3). Approximately 80% of the genes from each GS were involved in the listed ontology terms. The top ranked GS1 and GS2 GO terms were mainly wounding and inflammation, which are typical SMC responses to surgical injury *in vivo* and TNFα or IL-1β stimulation *in vitro* ^20^. In contrast, the highest ranked term for GS3 and GS4 was apoptosis. It is well documented that ECs are highly sensitive to insults caused by TNFα or IL-1β because of apoptosis activation^29^.

Therefore, these data are highly consistent with previously reported experimental results, authenticating our experimental system and bioinformatic tools. More importantly, the pathways and GO terms summarized here implicate functional associations among the genes subject to differential regulations in SMCs versus ECs.

### Analysis of gene interaction networks (GINs) identifies common hubs and common bottlenecks in SMC (or EC)-enriched gene sets

**Figure 3:**
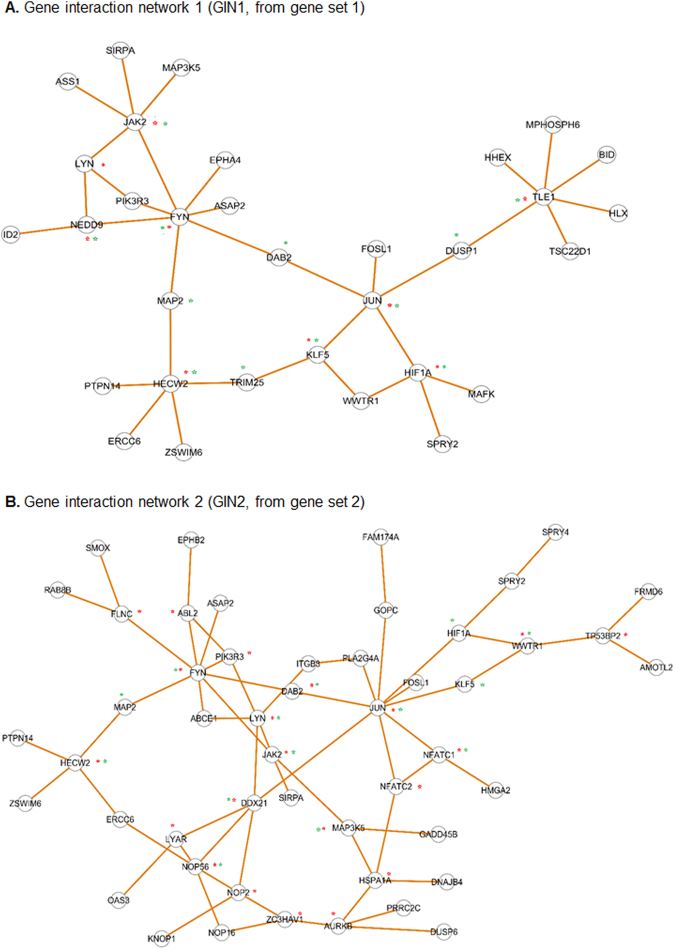
Analysis of SMC-enriched gene interaction network. (**A**) Gene interaction network 1 (GIN1) was derived from gene set 1 (GS1) which represents the genes strongly up-regulated in SMCs but down-regulated or slightly up-regulated in ECs after treatment with TNFα. (**B**) GIN2 was derived from GS2 which represents the genes strongly up-regulated in SMCs but down-regulated or slightly up-regulated in ECs after treatment with IL-1β. Hubs and bottlenecks are marked by red stars and green stars, respectively.

**Figure 4:**
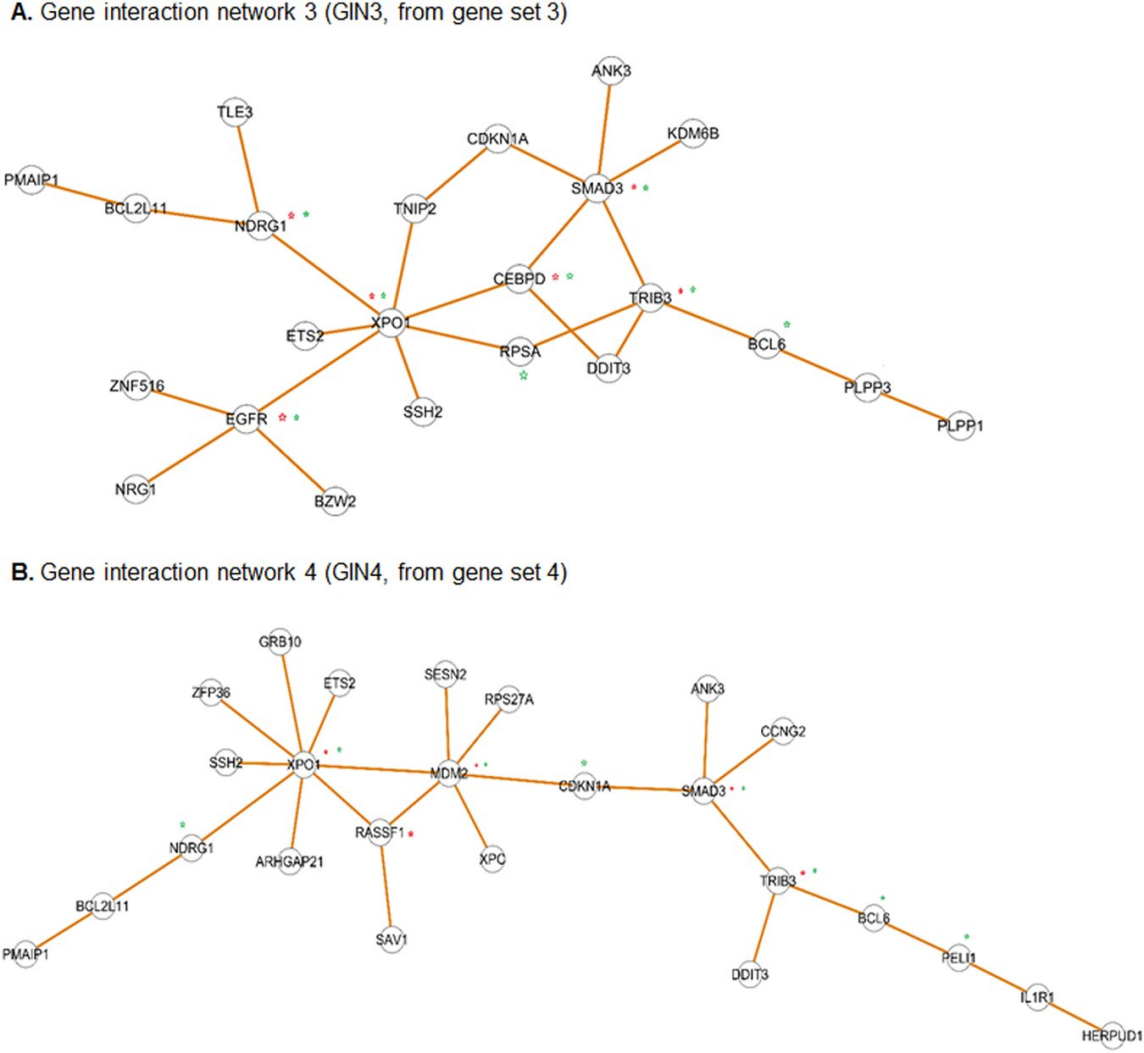
Analysis of EC-enriched gene interaction network. (**A**) GIN3 was derived from GS3 which represents the genes strongly up-regulated in ECs but down-regulated or slightly up-regulated in SMCs after treatment with TNFα. (**B**) GIN4 was derived from GS4 which represents the genes strongly up-regulated in ECs but down-regulated or slightly up-regulated in SMCs after treatment with IL-1β. Hubs and bottlenecks are marked by red stars and green stars, respectively.

We further investigated the functional relationships of the genes in the SMC-enriched or EC-enriched GSs by analyzing gene interaction networks (GINs). Using the esyN program to retrieve (from HumanMine database) information of gene functional relationships, e.g. regulation, co-expression^30,31^, we constructed four GINs: GIN1, GIN2, GIN3, and GIN4, corresponding to GS1, GS2, GS3 and GS4, respectively (Figs 3 and 4).

In each GS, approximately 25% of genes were included in the GIN. There are 32 nodes and 35 edges in GIN1, 50 nodes and 62 edges in GIN2, 23 nodes and 25 edges in GIN3, and 25 nodes and 25 edges in GIN4 (Table 1). The respective GINs (1 and 2) of SMC-enriched GS1 and GS2 exhibit largely similar structures (Fig. 3). The GIN structures of EC-enriched gene sets (GS3 and GS4) are very similar (Fig. 4).

**Table 1:** Hub and bottleneck genes identified based on the statistics of the gene interaction networks (GINs) including the numbers of nodes, edges and betweenness values (BET).

Number of nodes (genes) and edges (interactions)	GIN1		GIN2		GIN3		GIN4	
	Nodes	edges	Nodes	edges	Nodes	edges	Nodes	edges
	32	35	50	62	23	25	25	25
	Gene	edges	Gene	edges	Gene	edges	Gene	edges
Hub genes and numbers of edges (interactions)	FYN	7	JUN	9	XOP1	7	XOP1	8
	JUN	5	FYN	8	SMAD3	5	MDM2	6
	TLE1	5	LYN	5	TRIB3	4	SMAD3	4
	JAK2	5	DDX21	5	EGFR	4	RASSF1	3
	HECW2	4	NOP56	5	NDRG1	3	TRIB3	3
	KLF5	3	JAK2	4	CEBPD	3	IL1R1	3
	HIF1A	3	DAB2	4				
	NEDD9	3	NOP2	4				
	LYN	3	AURKB	4				
			HSPA1A	4				
			TP53BP2	3				
			HECW2	3				
			MAP3K5	3				
			ABL2	3				
			NFATC1	3				
			NFATC2	3				
			FLNC	3				
			LYAR	3				
			WWTR1	3				
Bottleneck genes and betweenness values (BET)	**Gene**	**BET**	**Gene**	**BET**	**Gene**	**BET**	**Gene**	**BET**
	JUN	1	JUN	1	XPO1	1	MDM2	1
	FYN	0.951	FYN	0.651	TRIB3	0.398	XPO1	0.869
	DAB2	0.629	DDX21	0.543	EGFR	0.376	SMAD3	0.801
	DUSP1	0.568	JAK2	0.37	NDRG1	0.37	CDKN1A	0.767
	TLE1	0.53	LYN	0.33	SMAD3	0.323	TRIB3	0.563
	JAK2	0.36	DAB2	0.319	CEBPD	0.295	BCL6	0.358
	HECW2	0.383	WWTR1	0.288	RPSA	0.288	PELI1	0.25
	KLF5	0.294	NOP56	0.239	BCL6	0.251	NDRG1	0.25
	HIF1A	0.263	HECW2	0.198				
	MAP2	0.223	MAP3K5	0.184				
	TRIM25	0.22	KLF5	0.18				
	NEDD9	0.144	NFATC1	0.18				
			HIF1A	0.18				
			MAP2	0.153				

Note: Nodes represent genes and edges refer to interactions. The betweenness of a node v is obtained by counting the number of all shortest paths, connecting any pair of nodes within the network, which are going through that particular node v. The value is divided by the number of all shortest paths connecting two nodes. The higher the betweenness value of a node, the more important the node in the network. A node with top ranked betweenness value is the key node in the network.

We next identified hub genes and bottleneck genes in the GINs, marked by red and green stars respectively in Figs 3 and 4. The hubs and bottlenecks are considered important centrality indices because they are major intersections between clusters in the network. If they are removed, the network collapses^23^. Hubs are ranked by node degree, or the number of interactions associated with a given node; bottlenecks are ranked by betweenness centrality. Since hubs and bottlenecks are distinctly defined and independently identified, they are not necessarily related. However, they may overlap in the same GIN as both are central genes based on their definitions. As listed in Table 1, FYN and JUN are top ranked hubs with 7 and 5 edges respectively in GIN1 (and similarly in GIN2); XPO1 and SMAD3 are top ranked hubs with 7 and 5 edges respectively in GIN3 (and similarly in GIN4). Interestingly, JUN and FYN are also top ranked bottlenecks with betweenness values of 1.00 and 0.95 respectively in GIN1 (and similarly in GIN2); XPO1 and SMAD3 are bottlenecks that both ranked high in GIN3 and GIN4.

We further observed that a number of hub genes were shared by GIN1 and GIN2, i.e., JUN, FYN, JAK2 and HECW2, and so were bottleneck genes including JUN, FYN, JAK2, DAB2, MAP2, HECW2, KLF5, and HIF1A. Similarly, GIN3 and GIN4 included common hub genes XPO1, SMAD3, TRIB3, as well as common bottleneck genes XPO1, SMAD3, NDRG1, BCL6, BCL2L11, and TRIB3. Some unique hub and bottleneck genes not shared between GINs were also identified (Table 1), e.g., hub genes LYN and NFATC1 and bottleneck genes SMAD9 and NEDD9.

Taken together, the results from TNFα and IL-1β treatments both reveal that a defined group of hub and bottleneck genes were highly up-regulated in SMCs, and another distinct group of hub and bottleneck genes were highly up-regulated in ECs.

### Common hub/bottleneck genes in SMC (or EC)-enriched GINs form common gene modules related to vascular disease

**Figure 5:**
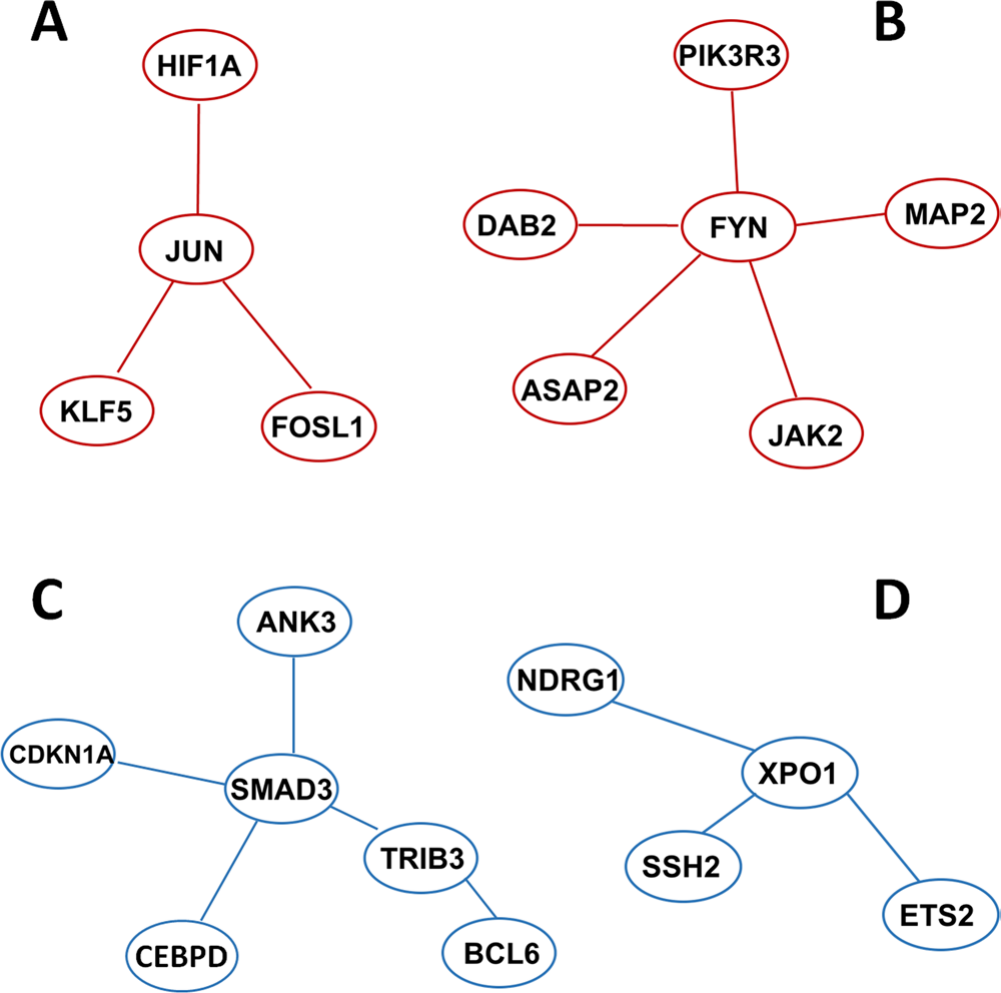
Gene modules extracted from hub and bottleneck genes. To identify a gene module, we selected a core node together with its nearest nodes in the GINs. Majority of the module genes are in the lists of common hubs or common bottlenecks (Table 1). The remainder of the genes were selected because of their close functional connectivity to the core nodes based on literature evidence.

Inspired by the foregoing results that common hub genes and common bottleneck genes were up-regulated by both TNFα and IL-1β in a SMC/EC or EC/SMC preferential manner, we further sought to identify gene modules which represent more centrality within GINs. A gene module is a subnetwork in a GIN formed by highly connected genes which are co-expressed in response to an environmental cue (e.g., cytokine)^32,33^. Using criteria based on this gene module definition, we identified two modules shared in SMC-enriched GIN1 and GIN2 and two common modules in EC-enriched GIN3 and GIN4. The criteria include: (1) A module is formed by a core node and its nearest nodes, (2) the module(s) is shared in both TNFα- and IL-1β-stimulated GINs, (3) the core node in the module is both a hub and a bottleneck, (4) the selection of each module gene is supported by literature evidence for its pathophysiological importance in vascular cells or relevant cell types. We thereby identified, in the SMC-enriched GINs, a core node JUN connected with its nearest nodes KLF5, HIF1A, and FOSL1 (hereafter termed JUN module) (Fig. 5A), and a core node FYN with its nearest nodes JAK2, ASAP2, MAP2, DAB2, and PIK3R3 (termed FYN module) (Fig. 5B). In a similar fashion, in EC-enriched GINs we identified the SMAD3 module including SMAD3 (core node), BCL6, CDKN1A, CEBPD, TRIB3, and ANK3 (Fig. 5C), and XPO1 module containing XPO1 (core node), ETS2, SSH2, and NDRG1 (Fig. 5D). Most of the genes in these four modules are hubs or bottlenecks. However, some non-hub/non-bottleneck genes (e.g., FOSL1) or genes not directly connected to the core node (e.g., BCL6) were also included because of their close functional relationship with the core node evidenced in the literature.

## Discussion

Current treatments for cardiovascular disease do not discriminate between SMCs and ECs, causing EC damage and predisposing patients to life-threatening thrombosis. It is thus critically important to understand differential pathogenic gene networks in the two cell types, for developing therapeutics to selectively mitigate SMC hyperplasia while protecting ECs. However, there has been no reported RNA-seq investigation of SMC-versus-EC differential transcriptomic responses to the same pathogenic stimulation. By analyzing combined transcriptomes of human primary SMCs and ECs, we investigated their differential transcriptomic dynamics induced by TNFα or IL-1β. We have thus identified gene modules that correspond to cytokine-stimulated regulations in these two cell types in a differential manner.

Our study design was optimized for systematic and accurate analyses of the differential transcriptomic responses to pathogenic stimulation in SMCs and ECs. First, for disease relevance, we used human primary SMCs and ECs cultured under uniform medium conditions. Second, to ensure the reliability and reproducibility of results, we performed two sets of experiments using two cytokines in parallel (TNFα and IL-1β), both well-established for stimulating pathogenic phenotype changes in SMCs and ECs. Third, we customized algorithms to analyze combined transcriptomes for identification of functionally relevant genes that are prominently responsive to cytokine stimulation in a SMC-versus-EC differential manner (Fig. 1). This strategy allowed us to identify defined groups of hub and bottleneck genes, which further revealed distinct gene modules enriched in SMCs and ECs respectively. Importantly, while previous cell physiology studies showed similar outcomes of TNFα and IL-1β treatments^27^, our results indicate that between TNFα and IL-1β experiments of the same SMC or EC cell type, large overlap exists in pathways, GO terms, GSs, GINs, hubs, and bottlenecks. Moreover, we were able to extract common gene modules that were sensitive to both TNFα and IL-1β and are relevant to vascular diseases as evidenced in the literature (discussed below). Taken together, a logical context based on existing biological data validates our experimental design and resultant bioinformatic outcomes.

Recently, it has been increasingly recognized that a number of genes involved in the same biological process may be coordinatively regulated in order to effectively respond to environmental disturbance^24,34^. A concept of gene module is thereby defined as functionally related, co-expressed genes whose transcription is controlled by shared gene regulatory networks. As such, gene modules are considered as a central mechanism in cellular responses to pathogenic cues, and their identification and characterization provide an important approach to understanding disease mechanisms and hence potential therapeutic targets.

Of particular interest, based on previous experimental reports, the gene modules identified in this study are closely related to vascular disease. For example, the proteins encoded by the JUN module genes (JUN, KLF5, FOSL1, and HIF1A) have all been reported to promote SMC hyperplasia while inhibiting SMC apoptosis. These effects contribute to the development of neointimal lesions that would ultimately re-occlude the vascular lumen^35–38^. Furthermore, experimental evidence suggests that increased levels or activities of these proteins induce pro-inflammatory phenotypes detrimental to ECs^39–41^. Therefore, the JUN module appears to provide ideal intervention targets, inhibition of which would attenuate SMC hyperplasia while protecting ECs. Indeed, our recent report suggests that keeping the Klf5 protein from up-regulation is a probable approach to attenuating SMC phenotype change and neointima formation while enhancing endothelial recovery^42^.

The genes in the FYN module are predominantly involved in cell motility or migration; some are also related to cell cycle progression. The proteins encoded by FYN and JAK2 have been shown to directly regulate SMC migratory capacity^43–45^. Interestingly, it was reported that up-regulation of p55γ (encoded by PIK3R3) could ameliorate neointima formation^46^. The functions of MAP2, DAB2, ASAP2 in SMCs remain unknown, yet their established pro-migratory roles in other cell types warrant future investigations^47–49^. Nevertheless, further investigation of the FYN module would uncover its potential for therapeutic targeting to effectively inhibit SMC migration without endothelium damage.

Herein found to be EC-enriched after cytokine stimulation, the SMAD3 module contains a panel of established markers for endothelium dysfunction. The Smad3 protein is a well-established initiator of epithelial/endothelial-to-mesenchymal transition (EMT), and there is strong evidence for that Smad3-mediated EMT in ECs greatly contributes to vasculopathies^28^. Encoded by CDKN1A, p21 is a component of the cell cycle repressor machinery, and increase of p21 inhibits the growth of ECs and also induces their senescence^50^. C/EBP-δ (CEBPD) represents indispensable mediator/adaptor proteins required for type I cytokines’ downstream signal transduction (e.g. NF-kB pathway)^51^, and are responsible for the pro-inflammatory phenotype of EC^52,53^. Bcl6 (BCL6) and Trib3 (TRIB3) are closely related with apoptosis and have been shown to promote cell death in EC and other cell types^54,55^. Ankyrin-G (ANK3), however, has been shown to be protective in endothelial physiology^56^.

Compared to the SMAD3 module, the other EC-enriched module (XPO1) is much less known in ECs. Ets2 (ETS2), a transcription factor involved in a wide range of signaling pathways, has been recently linked to cardiovascular disease for its prominent role in the inflammatory phenotype of ECs^57^. NDRG1 is widely involved in the regulation of cell differentiation and proliferation, and its role in promoting EC inflammation is well-documented^58^. As discussed above, the TGFβ/Smad3 signaling axis is well known for initiating EMT and endothelium dysfunction^28^. While the product of XPO1 is a nuclear exportation protein, SSH2-encoded protein is involved in actin re-organization^59,60^, both unexplored for their role in ECs. Thus, more studies are warranted to evaluate the significance of this module in EC dysfunction.

**Figure 6:**
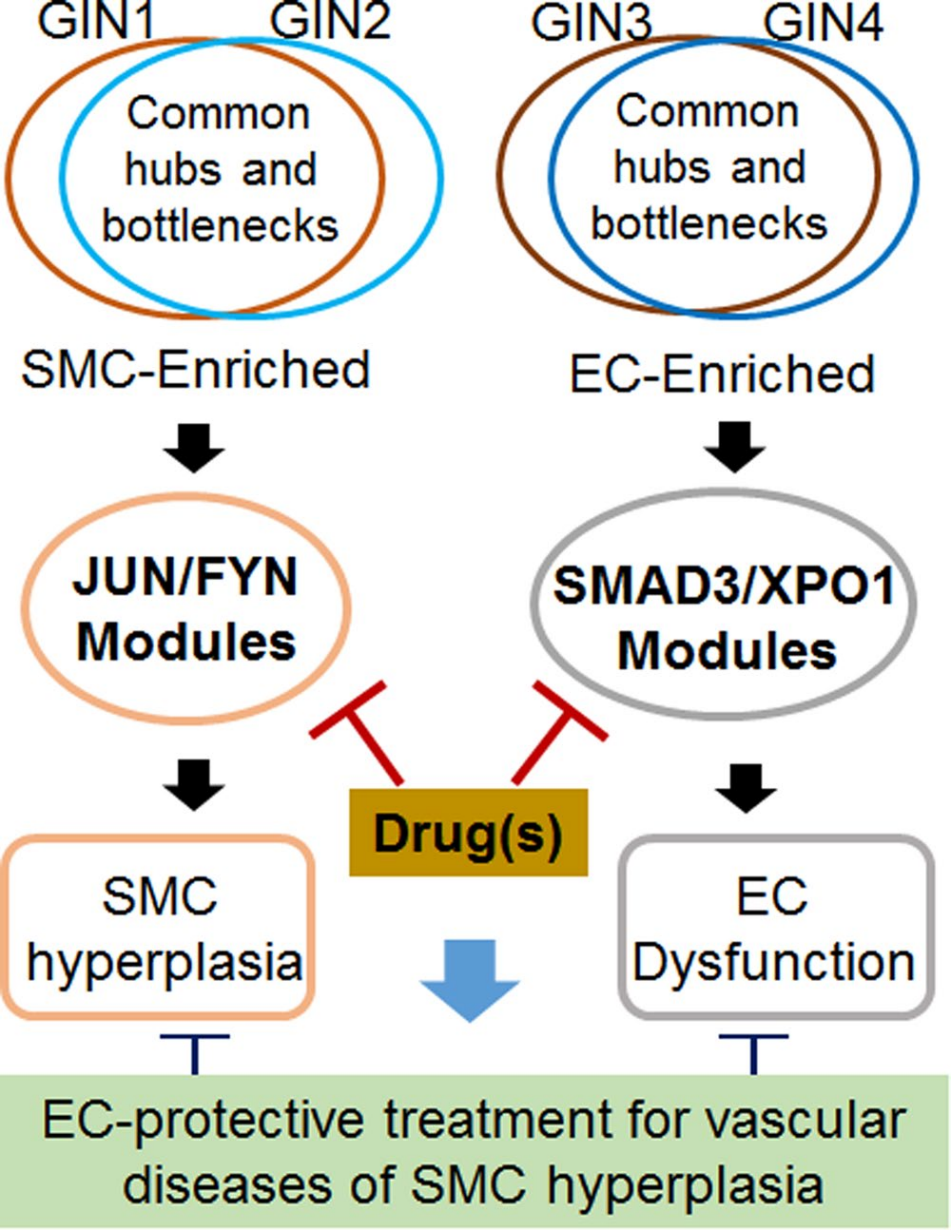
A schematic working model. Analysis of combined (SMC/EC) transcriptomic changes in response to pathogenic stimulation (TNFα and IL-1β treatments in parallel) led to the identification of common hubs and bottlenecks. These central nodes in GINs represent the key genes that were regulated differentially in the two cell types. Based on these genes, SMC-enriched JUN and FYN gene modules and EC-enriched SMAD3 and XPO1 modules were extracted which represent greater centrality. Therefore, these modules may provide interventional targets, inhibition of which could lead to safer treatments (low risk of EC damage) for vascular diseases associated with SMC hyperplasia such as restenosis.

Taken together, these four gene modules appear to represent the groups of central genes that are differentially regulated in SMCs versus ECs upon pathogenic stimulation. Moreover, in the literature, the majority of the genes are known important players involved in pathomechanisms of vascular diseases. As such, these modules may be targeted for the design and development of improved therapeutic strategies to selectively mitigate SMC hyperplasia while avoiding collateral damage to the endothelial protective inner lining (Fig. 6). Among these four modules, the core-node proteins c-Jun and Smad3 are “master transcription factors” that regulate the expression of an array of genes^61^. Thus, interesting questions arise as to whether c-Jun and Smad3 regulate FYN and XPO1 in SMCs and ECs respectively at the gene expression level or via protein interactions, and conversely, whether the Fyn kinase and XPO1 (exportin 1) modulate the activities of c-Jun and Smad3 respectively. Furthermore, given the prominent importance of c-Jun and Smad3 in their respective SMC and EC GINs, it is tempting to explore whether a combination therapy using inhibitors of these two target proteins produces favorable outcomes of simultaneously blocking neointimal growth and endothelial deterioration. Solving these questions awaits in-depth investigations via biological experiments.

## Conclusions

Our study provides the first comprehensive analysis of differential transcriptomic dynamics in human SMCs and ECs with a model of cytokine stimulation, which mimics the pathogenesis of restenosis and thrombosis following vascular injury. This analysis led to the identification of SMC-enriched as well as EC-enriched gene modules. These modules represent functionally clustered subnetworks highly responsive to pathogenic stimulation in a cell type-differential manner. Importantly, the pathophysiological significance of the majority of these genes is supported by literature evidence (discussed above). SMC hyperplasia and EC dysfunction are primary contributors to the major vasculopathies, including restenosis, thrombosis, and atherosclerosis. Therefore, continued research on gene modules identified herein would ultimately lead to better therapeutic strategies featuring selective mitigation of SMC pathophysiology without disturbing EC homeostasis.

### Data Availability

For information on GEO linking and citing, please refer to: https://www.ncbi.nlm.nih.gov/geo/info/linking.html. For GSE96962 study, please refer to: https://www.ncbi.nlm.nih.gov/geo/query/acc.cgi?acc=GSE96962.

## Electronic supplementary material

Dataset 1

dataset-2a

dataset-2b

Dataset-3a

Dataset-3b
