# Supplementary material for: Analysis of Combined Transcriptomes Identifies Gene Modules that Differentially Respond to Pathogenic Stimulation of Vascular Smooth Muscle and Endothelial Cells
Source: Sci Rep. 2018 Jan 10;8:395. doi: 10.1038/s41598-017-18675-2 (PMC5762668; doi:10.1038/s41598-017-18675-2)
Supplement: Supplementary file 1 — Dataset 1 [file 41598_2017_18675_MOESM1_ESM.doc]

**Analysis of Combined Transcriptomes Identifies Gene Modules that Differentially Respond to Pathogenic Stimulation of Vascular Smooth Muscle and Endothelial Cells**

Xiaokang Pan^1,2#^, Bowen Wang^1,3#^, Tiezheng Yuan^1^, Mengxue Zhang^1,4^, K. Craig Kent^1,3^, and Lian-Wang Guo^1,4^,*

^1^Department of Surgery, Wisconsin Institute for Medical Research, University of Wisconsin School of Medicine and Public Health, Madison, WI 53705, USA

^2^James Molecular Laboratory, Comprehensive Cancer Center, The Ohio State University, Columbus, OH 43240, USA

^3^Department of Surgery, College of Medicine, The Ohio State University, Columbus, OH 43210, USA

^4^Department of Surgery and Department of Physiology & Cell Biology, Davis Heart and Lung Research Institute, The Ohio State University, Columbus, OH 43210, USA

*Short title:Transcriptome analysis of smooth muscle and endothelial cells*

^#^These authors contributed equally to this work.

*Corresponding author:

Lian-Wang Guo, Ph.D.

Associate Professor, The Ohio State University

Davis Heart and Lung Research Institute

Department of Surgery, Department of Physiology and Cell Biology

473 W. 12th Avenue, Columbus, OH 43210

Phone: 614-292-5276. Lianwang.Guo@osumc.edu

**Competing Interests:**The authors have declared that no competing interests exist.

**Supplemental Materials**

Figure S1

**A. TNFα-treated cells B. IL-1β-treated cells**


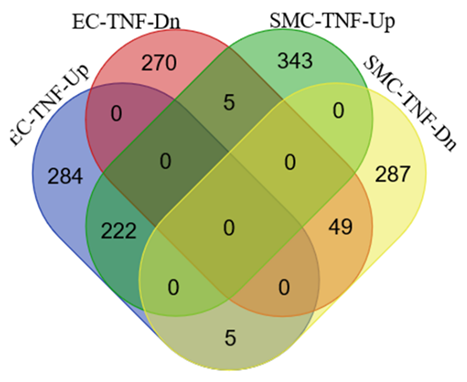

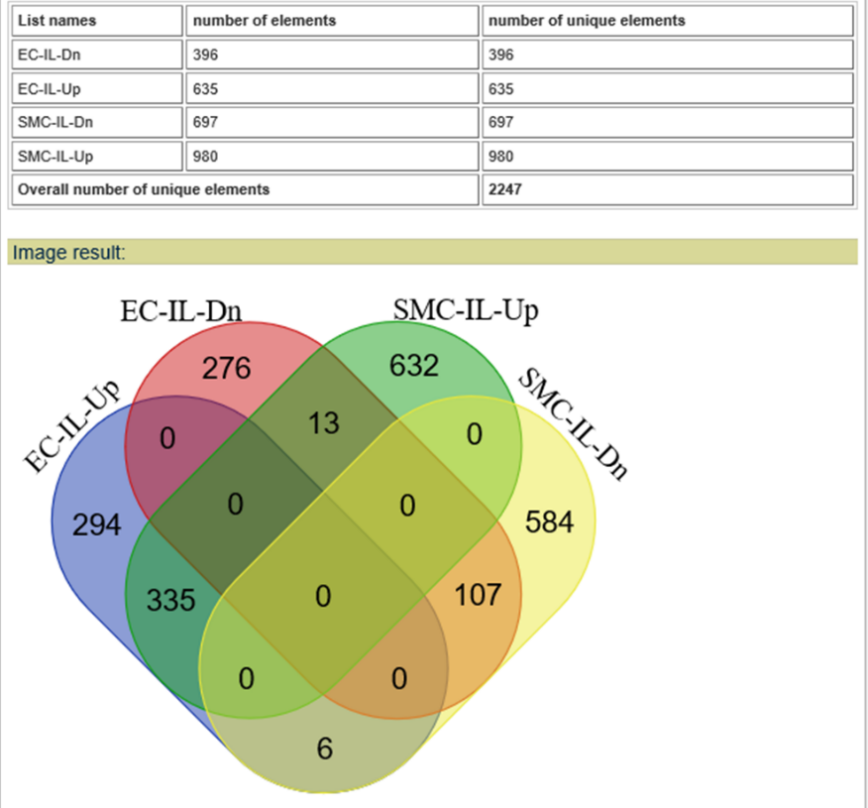


**Figure S1. *Venn diagrams revealing the genes regulated oppositely in SMCs and ECs following cytokine stimulation***

Human SMCs and ECs were cultured, and treated without or with TNFα (or IL-1β), prior to sample preparation for RNA-seq, as described in detail in Methods. A. TNFα-treated cells. B. IL-1β-treated cell. Up: genes up-regulated after cytokine treatment. Dn: genes down-regulated after cytokine treatment. Genes with fold change ≥2 (or ≤ 0.5) and adjusted p-value ≤0.05 were selected.

Figure S2

A. TNFα-stimulated


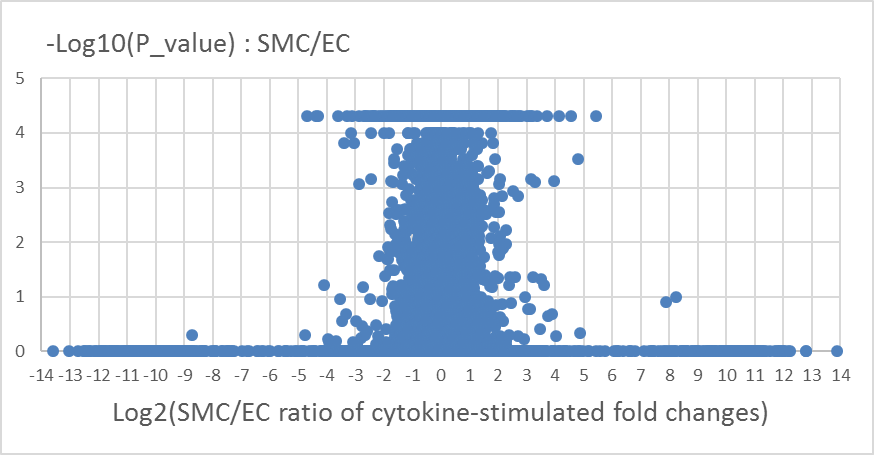


B. IL-1β-stimulated


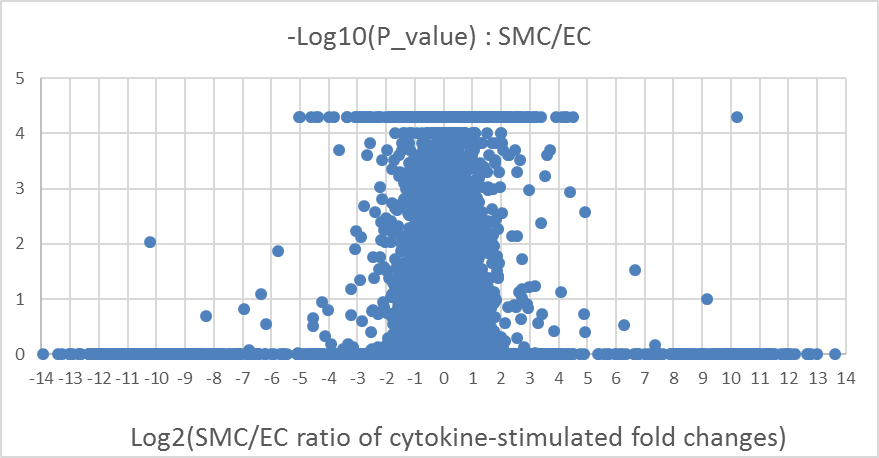


**Figure S2. *Vocano plot of adjusted P value versus the SMC/EC ratio of cytokine-stimulated fold changes***

Human SMCs and ECs were cultured, and treated without or with TNFα (A) or IL-1β (B), prior to sample preparation for RNA-seq, as described in Figure S1. The same set of data are presented in Table S2 and Table S3.

Figure S3


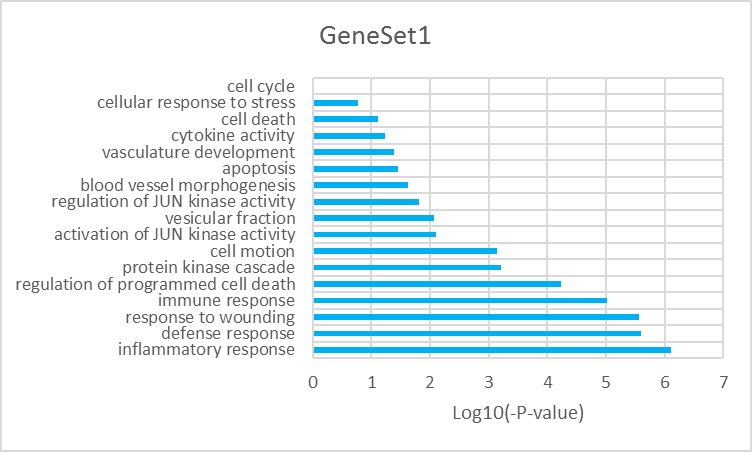


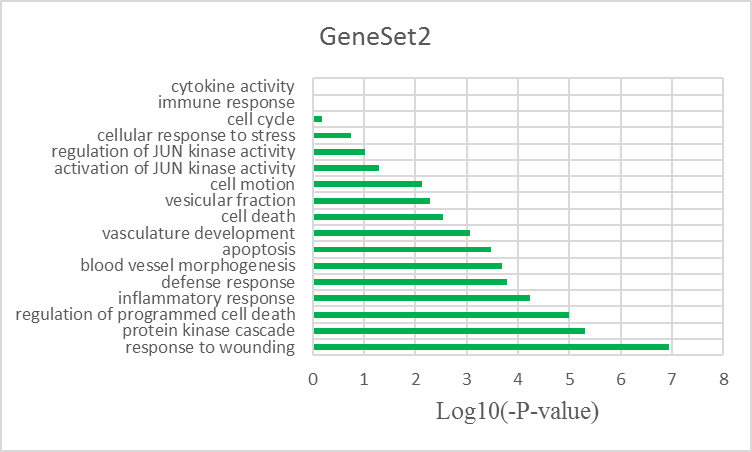


Figure S3 (continued)


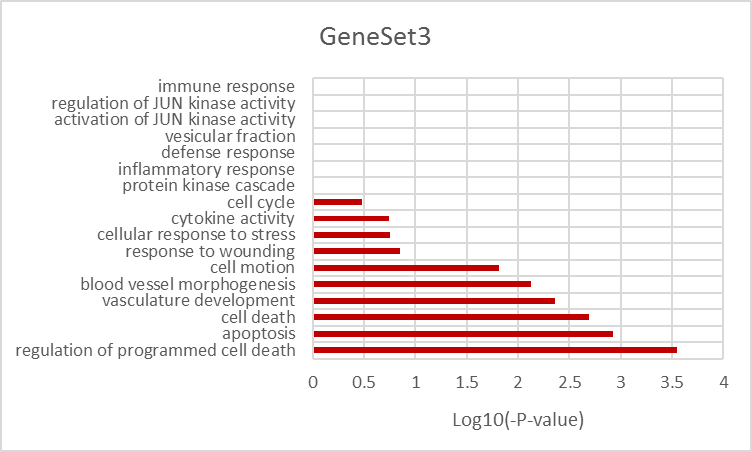


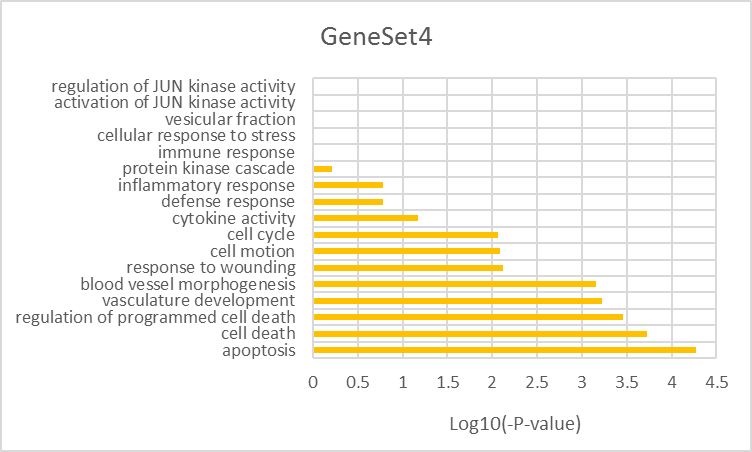


**Figure S3. Gene ontology enrichment analysis**

Human SMCs and ECs were cultured, and treated without or with TNFα or IL-1β, prior to sample preparation for RNA-seq, as described in Figure S1. Four gene sets were selected as listed in Table S2 and Table S3, which were used for gene ontology enrichment analysis.

**Table S1. Genes oppositely regulated in SMCs and ECs due to TNF-α or IL-1β stimulation** (corresponding to Figure S1).

|  | Change of gene expression | Oppositely regulated genes |
| --- | --- | --- |
| TNF-α | Up in SMCs but down in ECs | LYN, NHSL2, EVA1A, LYPD1, AMIGO2 |
|  | Down in SMCs but up in ECs | PDE5A, ZHX2, DHRS3, BCL2L11, BMF |
| IL-1β | Up in SMCs but down in ECs | CHST15, FGD4, ARID3A, GADD45B, DNAJB4, LYPD1, AMIGO2, ENC1, 4-Mar, NHSL2, EVA1A, RNF152, LMCD1 |
|  | Down in SMCs but up in ECs | SLC6A4, PDE5A, RGS2, CSRP2, E2F7, ANKRD33B |

**Table S4. KEGG-pathway enrichment analysis**

|  | **Pathway** | **Gene Count** | % | **Adjusted**  **P-Value** |
| --- | --- | --- | --- | --- |
|  |  |  |  |  |
|  | NOD-like receptor signaling pathway | 7 | 3.448275862 | 2.69E-04 |
|  | Toll-like receptor signaling pathway | 7 | 3.448275862 | 0.003521232 |
|  | Cytokine-cytokine receptor interaction | 11 | 5.418719212 | 0.004733028 |
|  | Chemokine signaling pathway | 8 | 3.9408867 | 0.01957032 |
| GS1 | Chondroitin sulfate biosynthesis | 3 | 1.477832512 | 0.040992755 |
|  | Jak-STAT signaling pathway | 6 | 2.955665025 | 0.077577149 |
|  | Epithelial cell signaling in Helicobacter pylori infection | 4 | 1.97044335 | 0.078216243 |
|  | B cell receptor signaling pathway | 4 | 1.97044335 | 0.098111225 |
|  |  |  |  |  |
|  | MAPK signaling pathway | 13 | 5.158730159 | 0.001449449 |
|  | NOD-like receptor signaling pathway | 6 | 2.380952381 | 0.003553099 |
|  | Viral myocarditis | 6 | 2.380952381 | 0.006359323 |
|  | Cytokine-cytokine receptor interaction | 11 | 4.365079365 | 0.011382418 |
|  | Wnt signaling pathway | 8 | 3.174603175 | 0.012620657 |
|  | Apoptosis | 6 | 2.380952381 | 0.01466903 |
|  | Cell adhesion molecules (CAMs) | 7 | 2.777777778 | 0.022401507 |
| GS2 | Natural killer cell mediated cytotoxicity | 7 | 2.777777778 | 0.023153946 |
|  | Toll-like receptor signaling pathway | 6 | 2.380952381 | 0.026254105 |
|  | Epithelial cell signaling in Helicobacter pylori infection | 5 | 1.984126984 | 0.026345456 |
|  | p53 signaling pathway | 5 | 1.984126984 | 0.026345456 |
|  | B cell receptor signaling pathway | 5 | 1.984126984 | 0.036037202 |
|  | VEGF signaling pathway | 5 | 1.984126984 | 0.036037202 |
|  | Jak-STAT signaling pathway | 7 | 2.777777778 | 0.044220295 |
|  |  |  |  |  |
|  | Cytokine-cytokine receptor interaction | 9 | 6.666666667 | 0.002789634 |
|  | Glycerophospholipid metabolism | 4 | 2.962962963 | 0.025776625 |
| GS3 | Ether lipid metabolism | 3 | 2.222222222 | 0.042609175 |
|  | ECM-receptor interaction | 4 | 2.962962963 | 0.044163123 |
|  | Glycerolipid metabolism | 3 | 2.222222222 | 0.06682978 |
|  |  |  |  |  |
|  | p53 signaling pathway | 6 | 3.03030303 | 0.00274188 |
|  | TGF-beta signaling pathway | 6 | 3.03030303 | 0.00787652 |
| GS4 | Cell cycle | 7 | 3.535353535 | 0.008684483 |
|  | Cytokine-cytokine receptor interaction | 10 | 5.050505051 | 0.011970198 |
|  | Ether lipid metabolism | 3 | 1.515151515 | 0.089304462 |
|  |  |  |  |  |
